# Deceptive Online Content Detection Using Only Message Characteristics and a Machine Learning Trained Expert System

**DOI:** 10.3390/s21217083

**Published:** 2021-10-26

**Authors:** Xinyu (Sherwin) Liang, Jeremy Straub

**Affiliations:** 1 School of Engineering, Technology, Mathematics and Sciences, Dallas College—North Lake, Irving, TX 75038, USA; e3472737@student.dcccd.edu; 2 Institute for Cyber Security Education and Research, North Dakota State University, Fargo, ND 58105, USA

**Keywords:** intentionally deceptive online content, fake news, message characteristics, machine learning trained expert system, social media

## Abstract

This paper considers the use of a post metadata-based approach to identifying intentionally deceptive online content. It presents the use of an inherently explainable artificial intelligence technique, which utilizes machine learning to train an expert system, for this purpose. It considers the role of three factors (textual context, speaker background, and emotion) in fake news detection analysis and evaluates the efficacy of using key factors, but not the inherently subjective processing of post text itself, to identify deceptive online content. This paper presents initial work on a potential deceptive content detection tool and also, through the networks that it presents for this purpose, considers the interrelationships of factors that can be used to determine whether a post is deceptive content or not and their comparative importance.

## 1. Introduction

Online social media interconnects the public, allowing personal news, photos, videos, and other content to be easily shared with friends, family, and anyone else who cares to read it. The reach of each individual’s or organization’s content is based on who chooses to read it directly and who chooses to re-share it. In this regard, social media sites have democratized news content. In the process, they have removed the filter of news media organizations—allowing content to flow unimpeded (and, in some cases, unedited and un-fact-checked) from writer to reader almost instantly.

This person-to-person communications capability allows for social progress. It facilitates members of the public banding together to demand the righting of wrongs. Events like the “Arab Spring” uprisings have demonstrated the power of social media coordination [1,2] (though some have minimized its role [3] or suggested social media use was a product of protests instead of a cause for them [4]). However, it has also provided a platform for those who seek to distribute misinformation. Some misinformation can be attributed to benign causes, such as different perspectives on an event or posting users themselves believing inaccurate information. In other cases, individuals and organizations post content knowing that it is wrong and do so with the intent to manipulate readers. Large-scale “misinformation network[s]” have been blamed for interference with the 2016 U.S. presidential election [5]. Misinformation has also been linked to the New Jersey “Bridge Gate” event [6] and even contributed to an armed standoff in the “Pizza Gate” incident [6,7].

Amongst its many consequences, Ognyanova, et al. [8] note that deceptive online content can reduce the public’s trust in traditional media and galvanize trust in political institutions based on readers political predispositions. A variety of potential solutions for responding to so-called “fake news” and mitigating the impact of intentionally deceptive online content have been proposed, ranging from ratings systems to warnings to blocking it [9]. However, these systems require a method to identify the deceptive content, in order to take whatever mitigation action that they propose. Strategies such as textual analysis [10,11], article characteristics analysis [12], and network analysis [13] have been proposed.

In many cases, neural networks [14] have been used as part of this analysis. However, this is problematic in its own right, as neural networks have been shown to produce inaccurate results [15], in some circumstances, and to be susceptible to targeted attacks against their decision-making logic [16,17]. Various “explainable artificial intelligence” (XAI) efforts have been proposed [15] to try to help humans understand, mitigate, and respond to these issues; however, explainability falls short of accuracy in decision-making. Artificial intelligence-based deceptive content identification techniques, thus, may themselves become a source of online misinformation.

In [18], a neural-network-like technique for training a network whose pathways are pre-defined (and, thus, not susceptible to learning non-causal or inaccurate associations) was proposed. In [18] (and further development presented in [19]), randomly generated networks, rules, and facts were utilized to demonstrate the technique and characterize its efficacy in a generalizable manner (as described in [20]). 

This paper extends on this prior work by presenting the initial work in the application of this technology to an application area: the challenge of intentionally deceptive online content detection. Its contributions, thus, include that it presents work using the machine learning-trained expert system [18] technology in a particular application area and that it evaluates the efficacy of a particular approach, of using only certain message metadata, to identify deceptive online content.

This paper continues, in Section 2, with a review of prior work that provides a foundation for the work presented herein. Section 3 and Section 4 present the design of the system used for testing and the study methodology, respectively. Section 5 and Section 6 discuss and analyze the different network configurations that were used for deceptive content identification and their results. Section 7 compares the results of the techniques used herein to prior work and Section 8 discusses system limitations, before the paper concludes (in Section 9) and discusses key areas of potential future work.

## 2. Background

This section presents prior work in several areas that provide the foundation for the work presented herein. First, the issues related to deceptive content and fake news are reviewed. Next, Section 2.2, Section 2.3, Section 2.4 and Section 2.5 present different strategies for identifying deceptive online content. Section 2.6 discusses the issues with using artificial intelligence techniques in this analysis. Finally, Section 2.7 presents the machine learning trained expert system that is used for the work presented herein.

### 2.1. The Danger of Fake News

Social media has removed limits of physical distance, increased convenience, and facilitated global communications. Twitter, in particular, has become a channel for news distribution for many traditional media outlets due to its short message format and ease of registration and use. It is also used for business promotion and political campaigning [12].

Because of these advantages, though, Twitter has been used to spread rumors and shape public opinion. In the 2020 election, for example, fake news messaging has been alleged to have led many voters to believe false statements, reducing their confidence in the American democratic system. An Indiana University survey indicated that more than 43 percent of respondents believed that counting machines overcounted Biden’s votes, and about 49 percent believed that mail-in ballots contributed to voter fraud [21].

The dangers of intentionally deceptive social media posts are not just political in nature. A recent attack on several celebrities’ Twitter accounts resulted in false posts, pretending to be the celebrities, which were used to steal $120,000 from their followers [22].

### 2.2. Preventing the Spread of Intentionally Deceptive Online Content

Multiple methods have been devised to identify intentionally deceptive online content and limit its spread. Techniques have been proposed for several types of false information content including propaganda, conspiracy theories, and hoaxes [23]. Preventing the spread of misinformation first requires its identification. Three prevailing strategies have found frequent use for this purpose: manual verification, web-based approaches, and semantic approaches. Manual verification may have accuracy benefits; however, because of the proliferation of fake news, manual verification mechanisms lack the capacity to keep up with the deceptive content, in most circumstances. 

Because of this, several automated approaches have been proposed. To effectively automate fake news detection, it is first necessary to understand how social media spreads on the web. According to Shu, et al. [24] there are three main dimensions to the spread of the Web on social media: the “content dimension”, the “social dimension”, and the “temporal dimension”. The content dimension, which Shu, et al. [24] call the “what” of the news article or post, is the association between different articles, posts and other media regarding the specific content of news posts [24]. The social dimension, which Shu, et al. [24] call the “who” of the news article or post, is the relationship between the publisher, distributor, and consumers of the news post [24]. Finally, the temporal dimension, which Shu, et al. [24] call the “when” of the news article or post, is the behavior of users in their posting and commenting over time [24]. Combining these three dimensions allows the different factors that are associated with media spread to be characterized. For media to spread rapidly and widely, it needs to cover a topic of interest to a community (or the general public) and be read and spread by users in the interested community. This process must happen rapidly for the content to gain a wide audience while it is still relevant and of interest to the community or general public.

### 2.3. Network Analysis

Network analysis, specifically, is a method of detecting fake news based on the properties of social networks. According to Shu, et al. [24], several properties make social networks a breeding ground for fake news. First, on social networks, it is easy to find people with similar views, so they are likely to form echo chambers. Second, there is the presence of individuals who are persuasive and those who gullible users reinforce the collective impressions of the community. Social identity theory [24,25] explains this phenomenon. This makes dispelling fake news even more difficult. Third, because social networks stratify users based on their interests, this can create a filter bubble. Finally, malicious accounts, some of which are “bots”, can influence users’ perspectives through frequent automated messaging.

Shu, et al. [24] used these dimensions to develop network representations of “mutual relations and dependencies” that were used to identify deceptive content. The social and temporal dimensions, in particular, form the basis of network analysis techniques. Key to this analysis and most types of analysis from a social dimension perspective is posting user identification (both whether the posting user is an automated bot or not and, in the case of a human posting user, who the individual is). To this end, Chu [12] proposes a method to determine whether posting users are humans, bots, or human-assisted bots. This approach is based on the analysis of three characteristics: the interval and periodicity of posts, whether posts contain “spam or malicious content”, and the posting user’s account properties. Additionally, several techniques have been proposed for detecting fake and bot-operated accounts. Cresci, et al., for example, have developed an optimized classifier [26], a DNA-based modeling “spambot group” identifier [27], and adversarial model spambot detector [28]. 

While posting user identification is helpful, it is not a complete solution to deceptive content identification. Several other network analysis-related techniques have been proposed for this. A technique proposed by Conroy, Rubin, and Chen [13] compares the text content of posts with a database to verify its truthfulness. Databases used for this purpose include public knowledge repositories such as DBpedia ontology and the Google Relation Extraction Corpus, as well as newly emerging fact-checking sites. 

A second approach analyzes the authenticity of posts based on the known author of the post or the user information associated with it. Rathore [29] uses a web analysis technique that considers the user’s domain name and psychological factors to achieve an 80% accuracy rate at deceptive content detection. Others, such as Gadek, et al. [30], have combined the use of a knowledge base with the analysis of posting user information.

### 2.4. Text Body Analysis

Another approach is to analyze the text itself, without comparison to an external news source, to determine its accuracy. Numerous techniques for textual analysis have been proposed [31]. These approaches are aligned with Shu, et al.’s [24] “content dimension”. 

A key advantage of this style of approach is that databases of content are not required for verification purposes. The lack of comparison searching also facilitates fast analysis. Known truthful and deceptive text are analyzed to identify linguistic patterns (such as word usage, n-gram and syntactic structure, semantic similarities, and rhetorical relationships between linguistic elements) associated with deceptive content [10]. Hancock’s method of analyzing individual words and n-grams (referred to as the “bags of words” technique) is very straightforward. The inflections and tenses used in the text are analyzed to determine whether the text is deceptive [11]. 

A number of text analysis approaches have been proposed—many of which incorporate artificial intelligence techniques. Smitha, et al. [32] compared the use of naive Bayes classifiers, convolutional neural networks, and support vector machine algorithms and concluded that neural networks and support vector machines were the most effective. Mahabub [33], similarly, compared eleven different methods, including naive Bayes classifiers, the k-nearest neighbors algorithm, the random forest algorithm, artificial neural networks, and logistic regression and identified three as performing the best: the multi-layer perception algorithm, logistic regression, and X-gradient boosting. 

Aldwairi and Alwahedi [34] compared Bayesian networks, logistic regression, naïve Bayes, and random tree algorithms and found that logistic regression performed the best, in terms of precision and tied with the random tree algorithm as best performing in terms of recall and the f-measure metric. However, the two Bayesian techniques performed best in terms of the receiver operating characteristic metric.

Kudarvalli and Fiaidhi [35] concluded that the logistic regression and support vector machine algorithms worked the best (with logistic regression outperforming support vector machines by 1%) out of the four they compared. The two outperformed naïve Bayesian classifiers and the long short-term memory technique. 

Singh [36] compared three different types of neural networks’ (basic artificial neural networks, convolutional neural networks, and recurrent neural networks) performance with the LIAR and Kaggle datasets using four different vector space representations. They found that the recurrent neural networks outperformed in many, but not all, cases.

Albahr and Albahar [37] compared random forest algorithms, naïve Bayesian classifiers, neural networks, and decision trees and concluded that the naïve Bayes approach worked the best. Ahmad and Ramasamy [38], alternately, compared neural networks, support vector machines, naïve Bayesian classifiers, and gradient descent and found that neural networks performed the best. 

While no single best text analysis approach algorithm has been identified, several promising results have been demonstrated. Techniques that combine multiple algorithms, such as Bonsu’s [39] combination of seven algorithms including logistic regression, support vector machines and decision trees, have also been proposed. This type of approach, through, suffers from limitations as it focuses on the usage of individual words as opposed to the overall semantics. Rubin [40] went beyond many of these techniques, by proposing a method called RST-VSM, which is based on analysis of rhetorical structures and discourse. 

### 2.5. Sentiment Analysis

An alternate approach, which goes beyond basic text analysis, is sentiment analysis. This approach focuses on the emotions of the text. The theoretical basis of this approach is that fake news authors often intentionally arouse the emotions of the readers to drive the success of their articles [10]. 

Sentiment analysis determines the type and intensity of the emotions expressed in text [10]. It is a branch of natural language processing which seeks to determine whether a text conveys objective or subjective information. If subjective information is identified, it is further assessed to determine whether it is presented in a positive, neutral, or negative manner, and whether it is expressed strongly or weekly. This technique is also referred to as opinion mining [10].

Sentiment analysis can provide additional information beyond what basic text analysis approaches are able to. Sharma, et al. [40], for example, note that positive sentiment tends to be exaggerated in positive fake comments as compared to real comments. Alternately, responses to fake news on social media tend to have negative sentiment. Given these patterns, sentiment analysis can be useful for detecting fake news. Anoop, et al. [41] showed how sentiment analysis could be valuable. They added sentiment analysis to a system designed to classify health news articles as true or false, which resulted in improved performance.

### 2.6. Artificial Intelligence Limitations and Explainability

Several of the previous subsections have described how artificial intelligence techniques, including a number of forms of neural networks, have been used perform fake news analysis. In addition to the overall accuracy numbers for each technique, a key consideration is whether techniques may suffer acute failures in terms of particular cases. For learning algorithms, like neural networks, this may be due to the algorithm learning invalid, non-causal associations. While certain associations may be true in many cases, they may not hold in all cases and thus cause bad assessments to be made in cases where they are inaccurate. Upadhayay and Behzadan [42] noted one issue with the LIAR dataset, which they corrected in the Sentimental LIAR dataset that is used for this work. The original included authors’ names, which could have resulted in the system forming truthfulness biases to certain particular names or names with similar characteristics. This could have resulted in prospective ethnic, gender, and other biases. Other potential biasing factors could also exist in data. For machine learning techniques that operate opaquely, exactly what is being learned by the system is unknown.

Transparency issues and system bias and learning concerns are known [43] to cause humans anxiety and have led to a number of groups raising concerns about automated decision making [44]. Concerns about their impact on vulnerable groups have led to some systems being poignantly termed “algorithms of oppression” by Noble [45] and “weapons of math destruction” by O’Neil [46]. Particularly problematic is a demonstrated correlation between strong performing AI systems and low explainability [47], though this correlation has not been shown to be causal. XAI techniques have been proposed in response to these problems. They are designed to help humans understand how systems are making decisions [47]. Fundamentally, XAI seeks to bring machine learning from being an opaque process to a fully transparent “glass box” [48].

### 2.7. Gradient Descent Trained Expert Systems

In response to the issues discussed in the previous subsection, a technique was proposed that goes beyond merely XAI. This technique, machine learning trained expert systems, which was introduced in [18,19], is used for the analysis performed in this paper. The technique is fundamentally different from the traditional neural network, in structure, though it provides conceptually similar machine learning capabilities. While neural networks are comprised of layers where each node in each layer is connected to each node in its neighboring layers, the machine learning trained expert system starts with the logical structure of a domain application-based rule-fact network and then performs machine learning to optimize the relationships (rule input contribution weights) between fact nodes. 

Facts store fractional values between 0 and 1, indicating the level of applicability of the fact statement. Rules have weights for their inputs (also between 0 and 1 and summing to 1) that determine the comparative impact of input rule values on the output rule. The system uses a specialized implementation of the gradient descent backpropagation technique to optimize the rule weightings based on a comparison of the output value of the system in its current form and the goal output value supplied during training. More details about the system used for this work are provided in Section 3. The network designs used are discussed in Section 5 and Section 6.

## 3. Experimental System Design

The experiments that are described in this paper were performed using a system derived from the one used for the experimentation presented in [18,19]. Unlike those papers, which used an ideal system (in some cases with perturbations) to train and test the gradient descent-trained expert system (as described in [20]), the work presented in this paper uses real world data from the Sentimental LIAR dataset [42] for supervised training (in place of the ideal system) and performance evaluation. However, the data storage and network implementation system used for the work herein is the same as was used in [18,19] as are the training and presentation-for-evaluation mechanisms.

**Figure 1:**
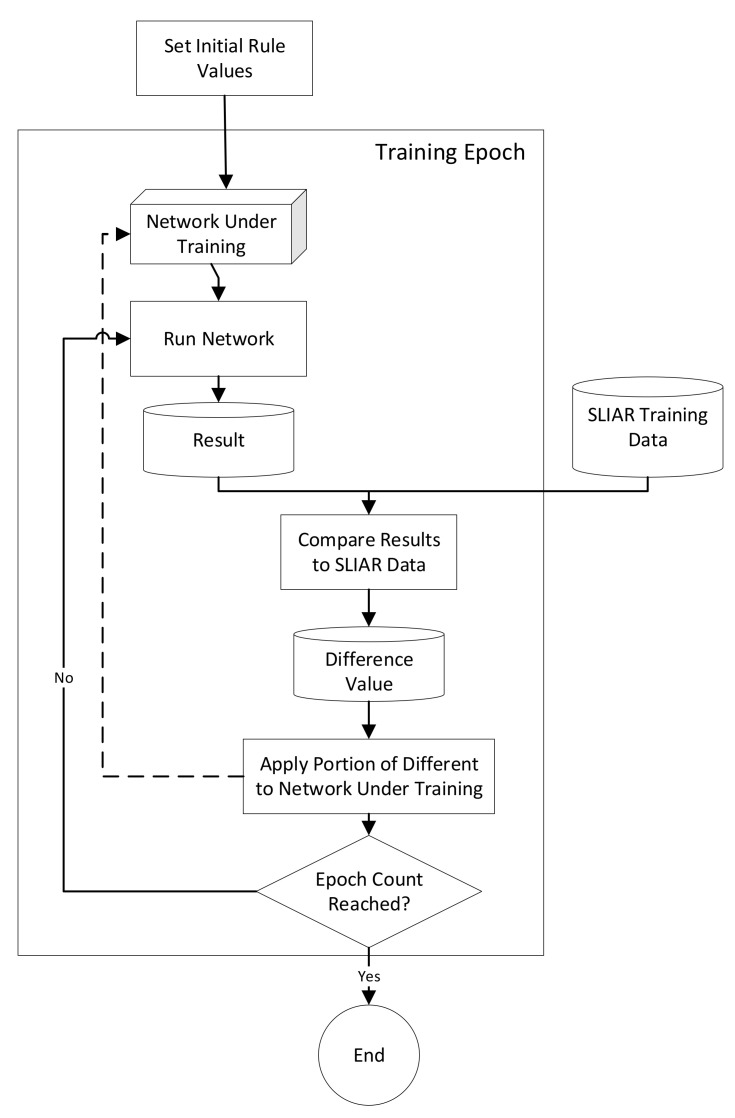
Training Process Using SLIAR Dataset (modified from [18]).

The training process that was used is presented in Figure 1. An initial network design was created for each test (twelve designs, in total, were evaluated). These designs are described in Section 5. The network was then trained using the process depicted in Figure 1, which determines the difference between the results of the network-under-training and the target result from the training data and distributes a portion of the difference to each rule that contributes to the output fact’s value (which is indicated with the dashed line in Figure 1). The training process is run for a given number of training records and epochs of training and the amount of difference that is distributed to the rules during each iteration is based on a specified velocity value.

After the velocity value is used to determine the amount of the difference to distribute, the level of contribution of each rule to the output fact must be determined, as the difference correction is distributed proportionately to the contribution of each rule. The contribution of each rule, *C_i_*, to the target fact, is determined using the equation [18]:

(1) $$C_{i}=W_{i}\times \underset{\left\{APT\right\}}{\prod }W_{R\left(m,h\right)}$$

where *W_i_* is the weighting for rule *i*, *W_R(m,h)_* is each rule’s weighting (*m* represents the rule and *h* represents the given weight value) and {*APT*} is the set of all of the rules that are passed through for the contribution. Note that rules that only the highest value will be used for a rule that is part of multiple rule-fact chains to the output fact.

**Figure 2:**
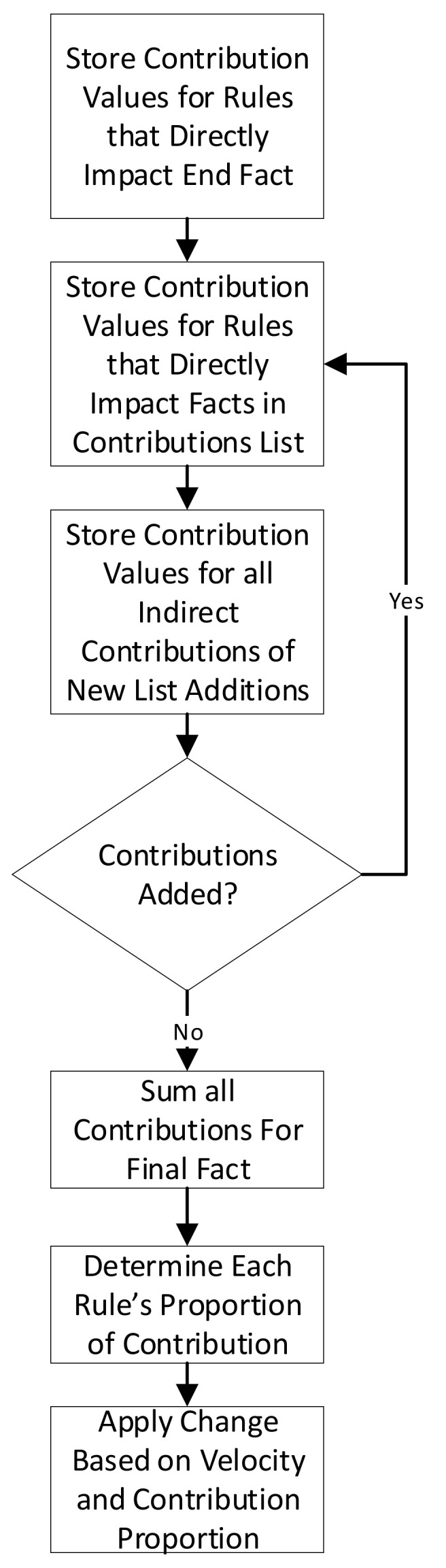
Node Change Determination Algorithm [18].

The difference value that is applied to a given rule weighting, *D_i_*, is determined by dividing the contribution of the rule is by the sum of all rules’ contributions. This is multiplied by the velocity value and the amount of difference that is being distributed. It is computed with the equation (modified from [19]):

(2) $$D_{i}=\frac{C_{i}}{\sum_{\left\{AC\right\}}C_{i}}\times V\times \frac{\left|R_{DS}-R_{T}\right|}{MAX\left(R_{P},R_{T}\right)}$$

where {*AC*} is the set of all rules that contribute to the output fact, *R_DS_* is the result from the training dataset, *R_T_* is the result from the network-under-training, *V* is the velocity and *MAX* is a function which returns the largest of the values passed to it. The process for applying the difference is depicted in Figure 2.

## 4. Methodology

This section describes the methodology used for the experimentation which is presented herein. Section 4.1 introduces the Sentimental LIAR dataset. Section 4.2 describes the data pre-processing that was performed to place the data in the correct format for use in the gradient descent-trained expert system. Section 4.3 describes the pre-processing used to correct errors and omissions in the data. Finally, Section 4.4 discusses the evaluation process that was used.

### 4.1. Sentimental LIAR Dataset

For direct interpersonal communications, a variety of signals can indicate deception such as unnaturally concealing one’s emotions, shrugging and indifference [49]. With text-based online content, these signals of deception don’t exist, making the identification of deception more difficult, as it must be ascertained from the text and characteristics of the message itself. 

To facilitate research regarding using emotional characteristics for deceptive content detection, Upadhayay and Behzadan [42] created the Sentimental LIAR dataset, based upon the older LIAR dataset [50]. Sentimental LIAR extended the LIAR dataset by using the Google and IBM Watson natural language processing technologies. The Google API was used to determine the overall “attitude of the text”, while the IBM API [51] was used to analyze the emotional characteristics of texts and assign a value to each of five emotions: anger, fear, joy, disgust, and sadness.

Sentimental LIAR was initially created by Upadhayay and Behzadan [42] for classifying fake claims. In [42], they used a variety of techniques to attempt to identify deceptive content. Values from the original LIAR dataset and values computed from those values were used. These values were augmented with the IBM and Google API data. In addition to the natural language processing APIs, they also used the Bidirectional Encoder Representations from Transformer (BERT) system. In the current work, six values derived from this dataset (credibility score, sentiment score, emotion score, macroscopic score, five emotions, and three intermediate facts) are used to train the expert system to predict the truth or falsity of presented statements. The pre-computed values from the Google and IBM natural language processing APIs, which are included in the dataset, are used, but the BERT system is not.

The networks that were developed for the current work, which are discussed in more detail in Section 6, utilize a number of values calculated from the SLIAR dataset. 

The credibility score, for example, is a percentage of untruthfulness, based on the past statements of the author.It is calculated by dividing the number of mostly_true_counts for the author by the sum of the values of the five statement count variables: barely_true_counts, false_counts, half_true_counts, mostly_true_counts, and pants_on_fire_counts. 

The sentiment score is designed to reflect the polarity of the text, with positive values showing positivity and negative values showing negativity. The sentiment value is computed from the five emotion values that were previously discussed.

### 4.2. Data Processing

The data format requirements of gradient descent trained expert system require that the data in the Sentimental LIAR dataset be processed before it can be used. In some cases, the required format change is simply a change to data presentation: for example, the scores for the five emotions (anger, fear, joy, disgust and sad) must be formatted into a 000.000 format. Additionally, since the system does not accept negative numbers and there are positive and negative sentiment scores, it was necessary to scale the sentiment scores so that they are all greater than or equal to zero. A method of scaling all numbers to the interval 0 to 1 is used, based on the equation:

(3) $$y=\frac{x-min}{max-min}$$

where *y* is the scaled score, *x* is the sentiment score in the dataset, and *min* and *max* represent the minimum and maximum sentiment scores, respectively.

Variables in the dataset that are text-based must also be converted into computable system-compatible numeric values. The label column, for example, is converted from six options (pants_fire, false, half-true, barely_true, mostly_true and true) to the values of 0.0, 0.1, 0.5, 0.6, 0.75, and 1.0. 

These values are used to assign values to other plain text variables. For each variable, each particular variable value is assigned the score (described above) of the average of all data records with that value. Variable values with less than 20 instances are assigned to the average of all records to avoid being overly influenced by potential outliers.

### 4.3. Data Cleaning

Like many datasets, the Sentimental LIAR dataset had a number of flaws. The pre-processing used to correct these issues discussed in Section 4.3.1 and Section 4.3.2.

#### 4.3.1. Incomplete/Blank Statistics

There are many blank values in the dataset (though these represent a small fraction—only about 6%—of the total data elements). The processing system is not designed to deal with missing input data, so it is necessary to preprocess the dataset to correct for missing values. A simple method for this correction was used where the mean of the values for the variable, in the entire data set, is used in place of missing values. This provides a neutral value for the facts (as using 1 or 0 would indicate data at an extreme and 0.5, while in the middle of the scale range, may deviate from the actual middle of the range of the data itself) that minimizes the impact of the missing data on the system’s decision making. Notably, this has a higher computational cost than using a pre-set value, as the average must be computed, and the ability to bypass missing data may be a valuable feature in a future version of the machine learning trained expert system software.

It is worth noting that the formula for the credibility score (which will be discussed in more detail in Section 5) uses the sum of all emotion scores as its denominator. Thus, cases where all emotion scores sum to zero are treated as blanks and processed in this manner.

#### 4.3.2. Corrective Processing

A few minor corrections were required to correct issues with some individual variables. Different expressions for the same state were consolidated. For example, “Washington D.C.”, “Washington, D.C.”, “District of Columbia”, “Washington DC”, and similar were consolidated (in this case, replaced with “DC”). Similarly, instances of capitalization differences and misspellings were corrected manually. Records with a blank value or a value of “None” were classified as “Unknown”.

### 4.4. Evaluation Techniques

This section presents the two evaluation methods used in this work. First, a method based on snapping is discussed. Then, a method based on thresholds is explained.

#### 4.4.1. Snapping Technique

The first form of evaluation was used to see how accurate the overall processing process is. This approach, in an ideal environment, would have data that was presented produce an output value that matched with its precise classification. For this to work, the data would need to not have significant errors or deviations in it (i.e., the training process would have to be able to operate effectively) and the rule-fact network would need to be normalized such that data is not shifted by passing through it. No attempt to normalize the network was made prior to this assessment. Thus, a high level of accuracy was not expected. Nonetheless, this assessment serves to illustrate the level corrective measures that are needed. 

The Sentimental LIAR dataset had five classifications for statements: pants_on_fire, false, barely_true, half_true, mostly_true, and true. Each statement was assigned a target value of 0.0, 0.1, 0.5, 0.6, 0.75, or 1.0, respectively, based on its categorization. To assess the uncorrected performance of the system, the system output value was compared to these values and “snapped” to the value that is closest to it (i.e., it is assigned the value that has the least level of difference to the true value). The snapped value was compared to the target value from the dataset and the percentage correct was recorded. 

#### 4.4.2. Threshold Method

This method is based on the approach used by Upadhayay and Behzadan [42] to assess the performance of several techniques they proposed for predicting the truthfulness of data in the Sentimental Liar dataset. They assigned each record in the data set a true or false value: “[1,0]” was used to indicate true and “[0,1]” was used to indicate false. The more granular classifications were placed into the true (true, mostly-true) and false (false, pants-fire, barely-true, half-true) classifications. Predictions were then assessed to see if they generated the correct true or false classification, since the other more granular classifications were inherently subjective.

To perform a similar analysis, a threshold between what is assessed to be true versus false must be determined. As was mentioned in the previous sub-section, the system is not expected to produce values that match the original scale, in all cases, without normalization. Given this, simply using 0.5 (as the half-way point on the scale) or 0.675 (half-way between 0.6 for barely-true and 0.75 for mostly-true) would not be expected to produce optimal results.

To determine the optimal threshold value, all values between 0.0 and 1.0 were assessed (at 0.01 increments), using the training data. The value with the highest accuracy for the training data was selected and used for processing (generation classification predictions for) the testing data set.

## 5. Network Design

This section presents the design and development of the rule-fact networks that were trained and used to classify the statements in the Sentimental LIAR dataset. The networks represent different logical configurations of the inputs for a phenomenon for which the exact logical relationships are not fully understood. Thus, through this exploration, not only is the best performing network identified for use, but a better understanding of the underlying phenomena is gained. 

### 5.1. Network Inputs and Facts

All of the networks use the same 12 inputs from the Sentimental LIAR dataset: anger, fear, joy, disgust, sad, subject, context, sentiment, state, party, credibility, and job. As the training process can effectively discount a given input, if needed, by reducing the weight given to it by the initial rule that processes it, subset combinations of inputs were not used. Additionally, due to their association with each other, the anger, fear, joy, disgust, and sad inputs are, in most cases, combined, early in the network, collectively becoming an emotion fact. Note that this emotion fact is different from the sentiment input, as the latter indicates the overall positivity or negativity reflected in the text. Table 1 discusses each of the 12 inputs and its relationship to deceptive online content identification. Table 2 presents sample data (note that the presented data are examples, not all possible values) from fields that have text-based data.

Beyond the input facts, intermediate facts are used to represent the resulting data from different relationships that have been created using the rule set. In several cases, multiple rules have been utilized to implement a complex rule, as the system only supports rules having two inputs. In these cases, the intermediate facts are used for processing purposes only. Though they have a specific meaning (i.e., the combination of their input elements), they are not necessarily results that could be separately analyzed and compared to a real-world phenomenon. The groupings’ output facts (such as the emotion fact described above) are designed to be potentially independently useful from the processing network and align with a real-world phenomenon (which may or may not be measurable, in each given case).

### 5.2. Network Rules

Rules define the relationships between the input facts, internal facts, and the fact or facts that serve as system outputs. The rule-fact networks (and, thus, the rules) are designed to associate logically related data. Rules are defined to associate inputs into summarizing facts (such as the previously described emotion fact) and to associate these summarizing facts with each other. When defining a network, it is important to note that oversimplification may be problematic, as it may prevent the ability to capture associations between different input or summarizing facts that are logical, but not exactly as expected. For example, it could be that one or several emotions are more associated with an output or are associated with an output along with another non-emotion fact. A larger and more nuanced rule network could more accurately capture a complex relationship like this. 

Thus, while a goal of the machine learning trained expert system is to ensure that rules represent logical, valid and causal relationships, this does not equate to networks that are necessarily very simple. Just like with neural networks, where performance can be significantly impacted by the number and configuration of the hidden layers, the rule network design is integral to system performance. Given the ability to include intermediate facts, there are literally an infinite number of networks that can be created. Potentially, this number could be constrained through the evaluation of inputs relative to each other. Inputs that are shown to act the same in all cases (presuming a complete set of use cases existed for an application) can be quickly grouped within the network design process, reducing the number of possible network designs significantly. Testing that shows a lack of correlating behavior or certain types of correlating behaviors could also be used to reduce the number of possible network implementations.

## 6. Network Implementations, Data Collection, and Analysis

Each of the following subsections describes a particular approach to the design of the rule-fact network and describes its performance in terms of the metrics discussed in Section 4. Following this, in Section 6.13, the performance of the different networks is compared. Then, in Section 6.14, the specific design processes used are discussed. Section 7 compares the networks’ performance to prior work with the LIAR and Sentimental LIAR datasets.

### 6.1. First Network Implementation and Results

**Figure 3:**
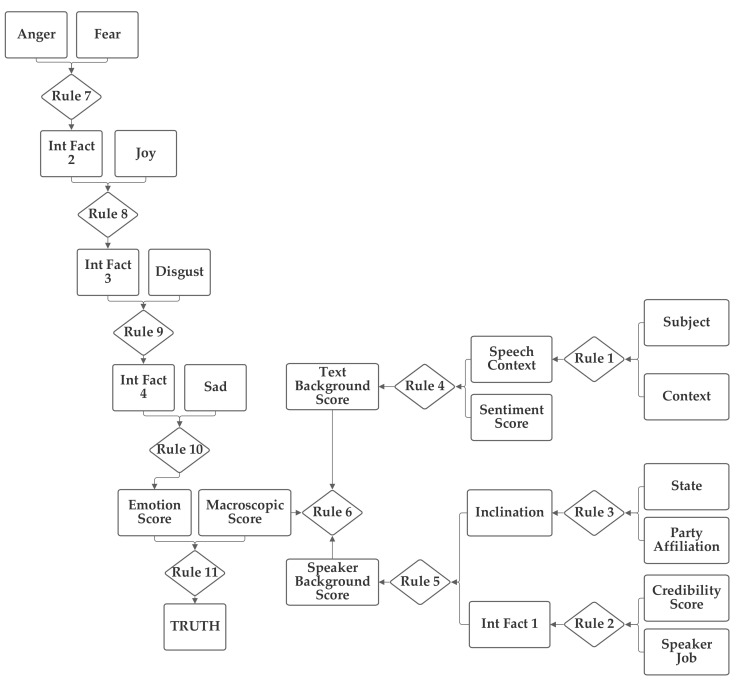
Depiction of Rule-Fact Network 1.

The first network configuration groups together the subject, context, and sentiment inputs, in one branch. The state and party inputs are grouped together in a second branch and the credibility and job inputs are grouped together in a third. The five emotion inputs are also used, separately, to compute the emotion score. The fact result of the emotion inputs, the emotion score, and the fact result of the other inputs, the macroscopic score, are then combined together by rule 11 to produce the truth output fact. This first network is presented in Figure 3. Note that this network makes use of intermediate facts. These facts are used to combine together multiple related facts that could logically serve as the inputs to a single rule, using several rules (as rules can have only two facts as inputs).

The network was trained with both 1 and 100 training epochs. As the results of the two levels of training are quite similar in most cases, the 100 training epochs results are discussed in Section 5.1. With 1 epoch of training (using the entire training portion of the 80% of the 12,836 Sentimental LIAR designated as the training subset), the first 1000 data records in the training subset were used to evaluate the normalization of the network. Of these, only 20.1% were matched to the correct one of the six categories without threshold normalization. The applicable threshold value was computed to be 0.11, using the data from the training dataset. With this threshold, it accurately classified 63.2% of the records in the training data subset. When this network and threshold were used with the testing data subset, it had an accuracy of 62.4%.

### 6.2. Second Network Implementation and Results

**Figure 4:**
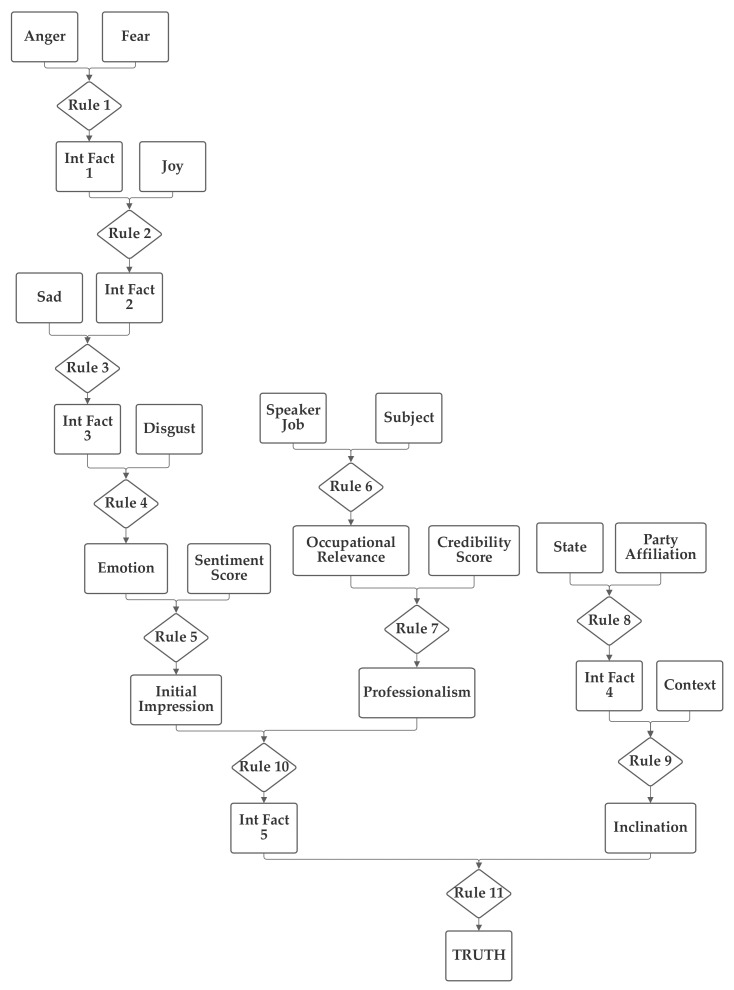
Depiction of Rule-Fact Network 2.

The second network places the emotion value (which is produced from the five component inputs) and the sentiment inputs together into one group related to the language understanding of the statement. The job, subject, and credibility inputs are then grouped together into a second professionalism group and the state, party, and context inputs are grouped together into a third inclination group. Through the use of an intermediate fact, the three are then brought together to produce the output truthfulness value. This second network is depicted in Figure 4.

As was performed with the first network, the second network was trained with both 1 and 100 training epochs, and the 1 epoch of training results (using the entire training portion of the 80% of the 12,836 Sentimental LIAR designated as the training subset) are now discussed. With the first 1000 data records in the training subset, the normalization was again evaluated. In this case, 21.3% were matched to the correct one of the six categories without threshold normalization. The applicable threshold value was again computed to be 0.11, using the data from the training dataset. With this threshold, it accurately classified 60.1% of the records in the training data subset. When this network and threshold were used with the testing data subset, it had an accuracy of 59.9%. Notably, this second network has a higher non-normalized matching accuracy; however, the performance with the threshold was lower than with the first network. 

### 6.3. Third Network Implementation and Results

**Figure 5:**
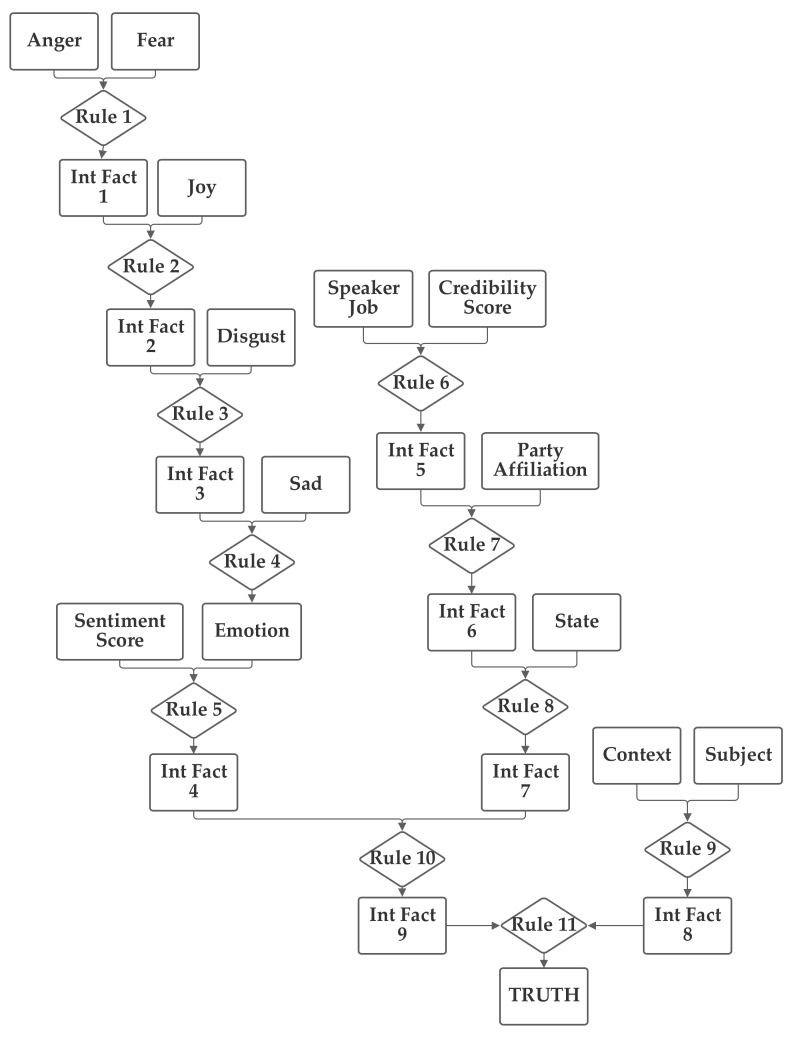
Depiction of Rule-Fact Network 3.

In the third network, shown in Figure 5, the emotion value, calculated from the five emotion-related inputs and the sentiment input are grouped together. The job, credibility, party, and state inputs are also grouped together. Finally, the context and subject are grouped together. This network investigates several groupings that do not have clear definitions to seek to identify relationships that may not be obvious to the network designer.

As was performed with the first three networks, the third network was trained with both 1 and 100 training epochs, and the 1 epoch of training results are now discussed. With the first 1000 data records in the training subset, the normalization was evaluated and 20.3% were matched to the correct one of the six categories without threshold normalization. The applicable threshold value was again computed to be 0.15, using the data from the training dataset. With this threshold, it accurately classified 63.2% of the records in the training data subset. When this network and threshold were used with the testing data subset, it had an accuracy of 62.4%. Notably, while this third network had a slightly higher non-normalized matching accuracy (20.3% versus 20.1%), the performance with the threshold was the same as the first network, both for the training data and the testing data. This shows how, in many cases, the training can optimize different networks to produce similar results, due to the applicability of the transitive property of multiplication. 

### 6.4. Forth Network Implementation and Results

**Figure 6:**
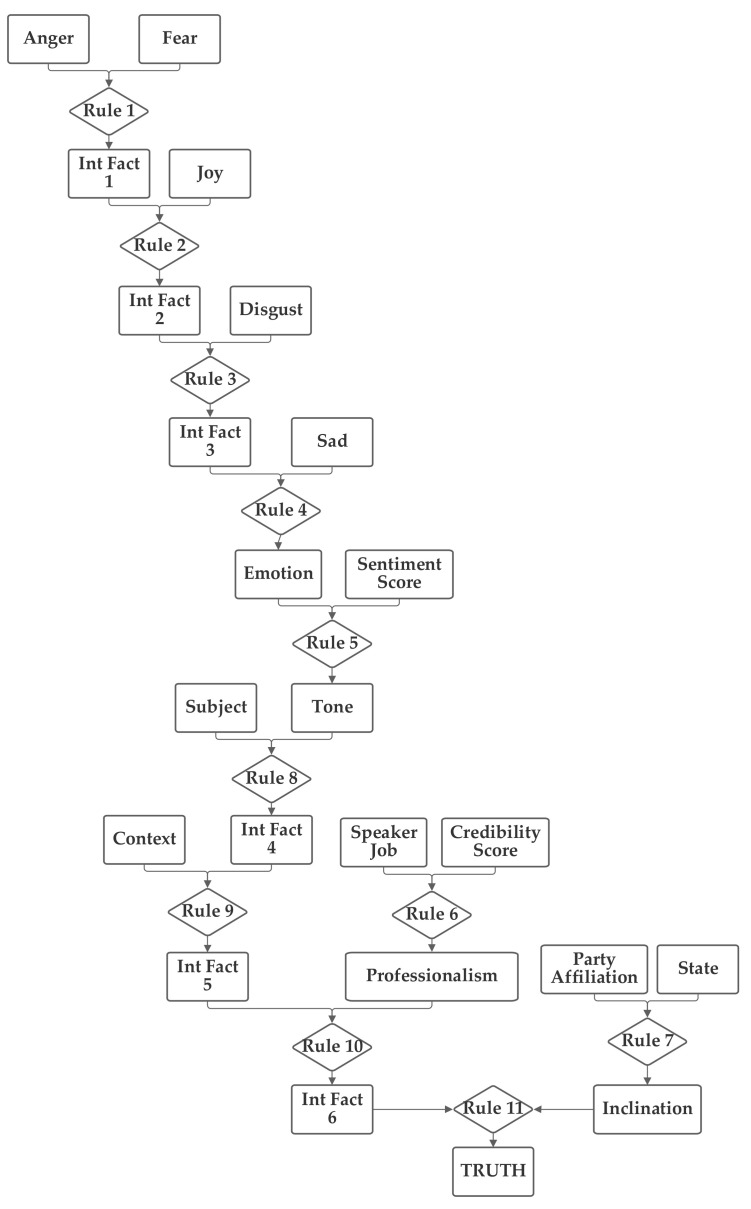
Depiction of Rule-Fact Network 4.

In the fourth network, the emotion value (computed from the five emotion-related inputs) and the sentiment, subject, and context inputs are grouped together. The job and credibility and party, and (separately) state inputs are also grouped together. The fourth network is presented in Figure 6.

Like with the previous networks, the fourth network was trained with both 1 and 100 training epochs, and the 1 epoch of training results are now discussed. With the first 1000 data records in the training subset, the normalization was evaluated and 19.7% were matched to the correct one of the six categories without threshold normalization. The applicable threshold value was again computed to be 0.11, using the data from the training dataset. With this threshold, it accurately classified 54.1% of the records in the training data subset. When this network and threshold were used with the testing data subset, it had an accuracy of 53.8%. This is the worst performing of the networks; it performed 8.5% worse (in absolute value), which is approximately 14% of the accuracy rate lower. This demonstrates that network design has a direct impact on performance and that it can produce issues that cannot be overcome by training (or, in some cases, may set training off on a path to producing an inferior result).

### 6.5. Fifth Network Implementation and Results

**Figure 7:**
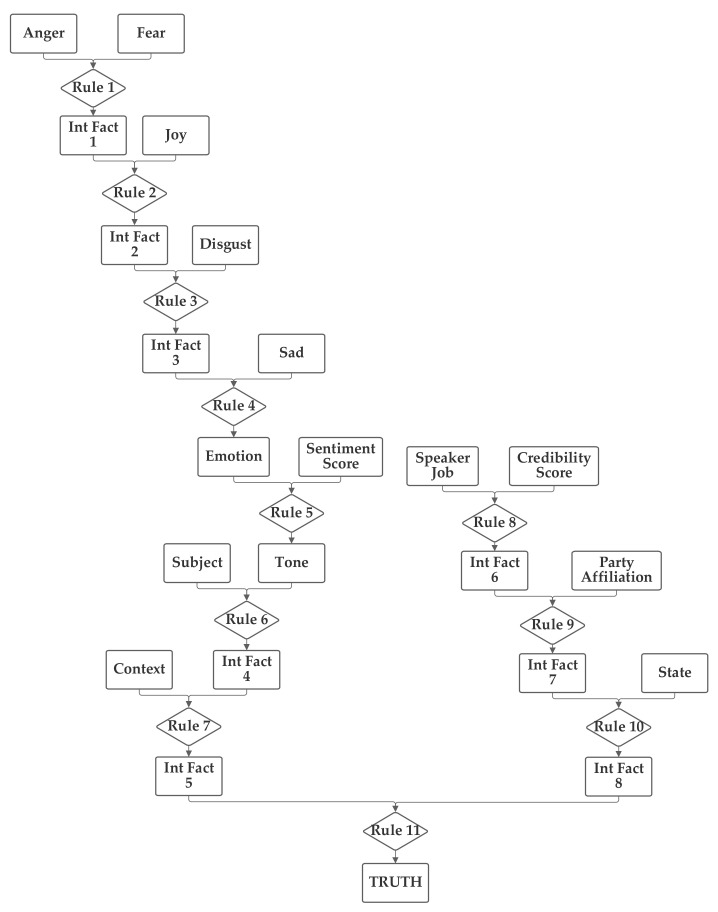
Depiction of Rule-Fact Network 5.

In the fifth network, the emotion value (produced from the five emotion-related inputs) and the subject, context, and sentiment inputs are grouped together. The job, credibility, party, and state inputs are grouped together in a second group. The fifth network is depicted in Figure 7.

Like with the previous networks, the fifth network was trained with both 1 and 100 training epochs, and the 1 epoch of training results are now discussed. With the first 1000 data records in the training subset, the normalization was evaluated, and 20.5% were matched to the correct one of the six categories without threshold normalization. The applicable threshold value was again computed to be 0.11, using the data from the training dataset. With this threshold, it accurately classified 60.7% of the records in the training data subset. When this network and threshold were used with the testing data subset, it had an accuracy of 60.3%. This result falls in between the best performance, evidenced by networks 1 and 3 and several others (which are discussed subsequently) and the worst overall performance, evidenced by network 4, demonstrating the responsiveness of performance accuracy, in some cases (which are not able to be overcome by training), to network design.

### 6.6. Sixth Network Implementation and Results

**Figure 8:**
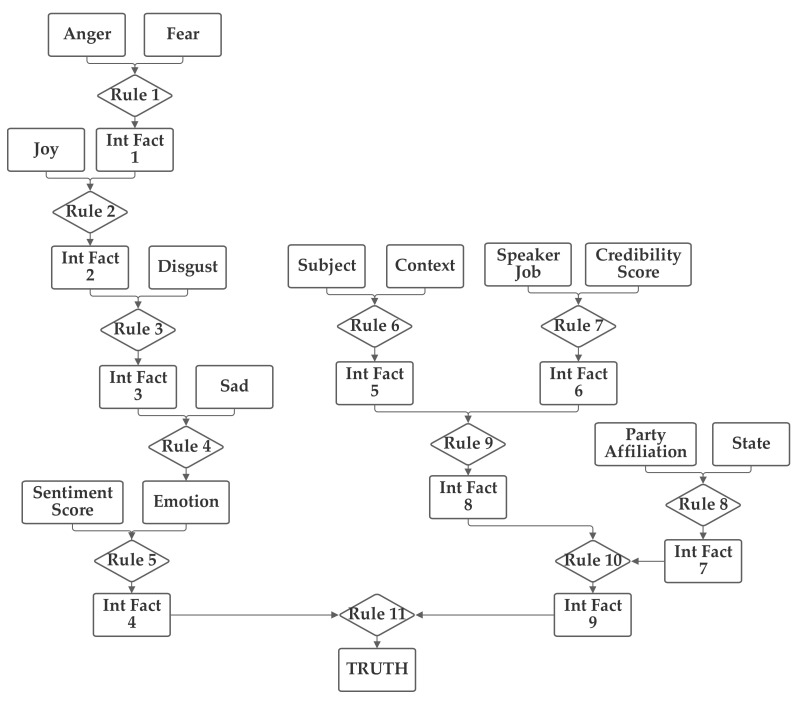
Depiction of Network 6.

In the sixth network, the emotion value (from the five emotion-related inputs) and the sentiment input are grouped together. The subject and context inputs are grouped together in a second group and the job and credibility inputs are grouped together in a third group. Finally, party and state are grouped together in a fourth group. The sixth network is presented in Figure 8.

The sixth network tied with networks 1 and 3 (and several subsequently discussed) to produce the highest accuracy level of 62.4%. Like with the previous networks, it was trained with both 1 and 100 training epochs, and the 1 epoch of training results are now discussed. With the first 1000 data records in the training subset, the normalization was evaluated and 21.7% were matched to the correct one of the six categories without threshold normalization. The applicable threshold value was again computed to be 0.11, using the data from the training dataset. With this threshold, it accurately classified 63.2% of the records in the training data subset. When this network and threshold were used with the testing data subset, it had an accuracy of 62.4%, again showing how training can produce, in some circumstances, similar optimization in different network designs. 

### 6.7. Seventh Network Implementation and Results

**Figure 9:**
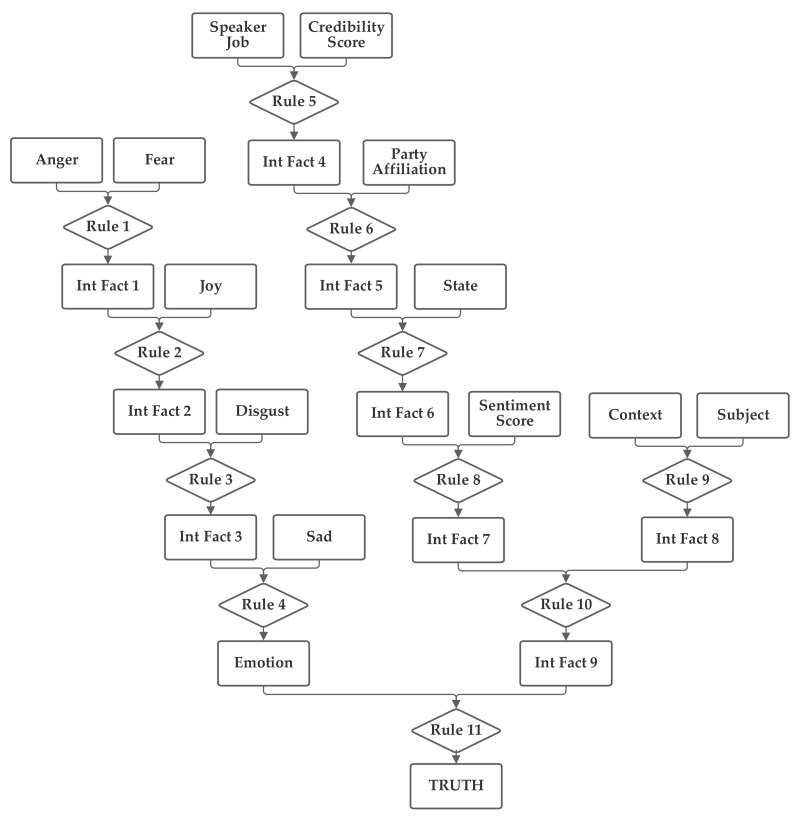
Depiction of Network 7.

In the seventh network, the job, credibility, party, state, and sentiment inputs are grouped together, and the context and subject are (separately) grouped together. The emotions value (based on the five emotion-related inputs) is brought together with these other values at rule 11, which produces the truthfulness output value. This network is presented in Figure 9.

The seventh network tied with networks 1, 3 and 6 (and three more subsequently discussed) to produce the highest accuracy level of 62.4%. Like with the previous networks, it was trained with both 1 and 100 training epochs, and the 1 epoch of training results are now discussed. With the first 1000 data records in the training subset, the normalization was evaluated and 22.3% were matched to the correct one of the six categories without threshold normalization. The applicable threshold value was again computed to be 0.14, using the data from the training dataset. With this threshold, it accurately classified 63.2% of the records in the training data subset. When this network and threshold were used with the testing data subset, it had an accuracy of 62.4%, again showing how training can produce, in some circumstances, similar optimization in different network designs. This network, in particular, demonstrates the importance of the threshold value for scaling, as it shows how different network configurations can alter the magnitude of the output of the truth value, while still producing similar logical results. 

### 6.8. Eighth Network Implementation and Results

**Figure 10:**
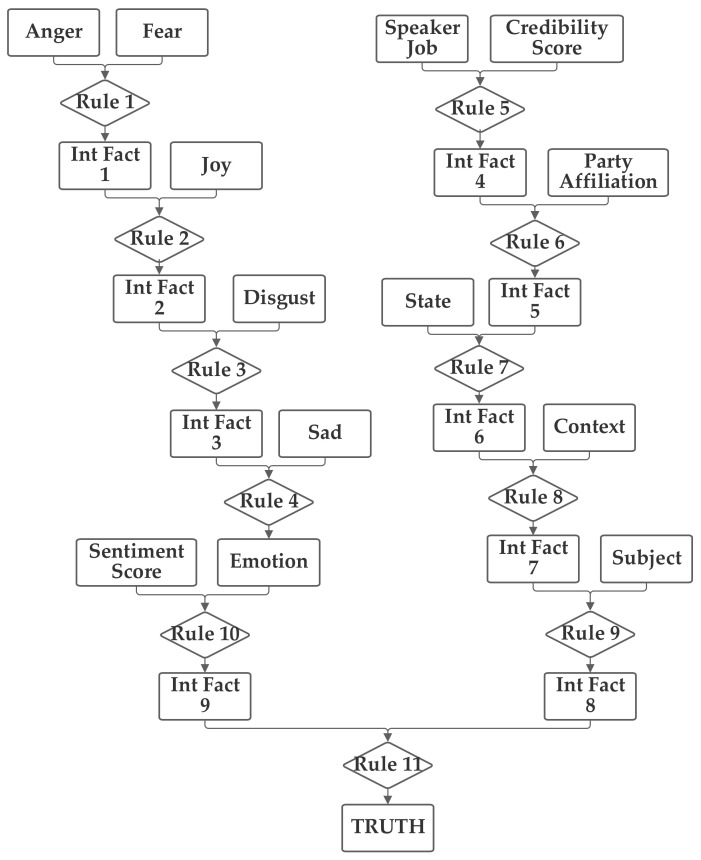
Depiction of Network 8.

In the eighth network, the emotion value (from the five emotion-related inputs) and the sentiment score input are grouped together. The remaining inputs (job, credibility, party, state, context, and subject) are grouped together in a second group. The eighth network is presented in Figure 10.

The eight network, similarly, tied with networks 1, 3, 6 and 7 (and two more subsequently discussed) to produce the highest accuracy level of 62.4%. Like with the previous networks, it was trained with both 1 and 100 training epochs, and the 1 epoch of training results are now discussed. With the first 1000 data records in the training subset the normalization was evaluated and 21.4% were matched to the correct one of the six categories without threshold normalization. The applicable threshold value was again computed to be 0.17, using the data from the training dataset. With this threshold, it accurately classified 63.2% of the records in the training data subset. When this network and threshold were used with the testing data subset, it had an accuracy of 62.4%, again showing how training can produce, in some circumstances, similar optimization in different network designs. This network, like the previous one, demonstrates the importance of the threshold value for scaling, as it shows how different network configurations can alter the magnitude of the output of the truth value, while still producing a similar logical result.

### 6.9. Ninth Network Implementation and Results

**Figure 11:**
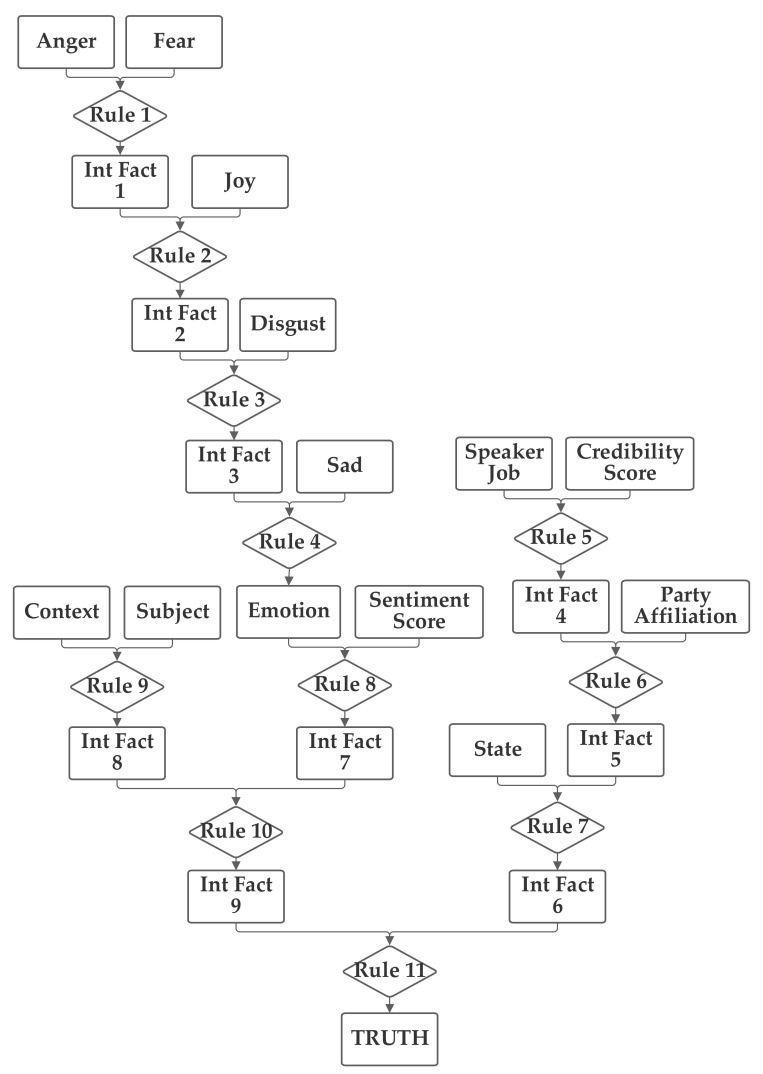
Depiction of Network 9.

The ninth network groups the emotion value (based on the five emotion inputs) and sentiment input together. The context and subject inputs are grouped together and the job, credibility, party, and state inputs are also (separately) grouped together. The ninth network is presented in Figure 11.

The ninth network tied with network 5 to produce a mid-range result. Like with the previous networks, it was trained with both 1 and 100 training epochs, and the 1 epoch of training results are now discussed. With the first 1000 data records in the training subset the normalization was evaluated and 20.5% were matched to the correct one of the six categories without threshold normalization. The applicable threshold value was again computed to be 0.11, using the data from the training dataset. With this threshold, it accurately classified 60.7% of the records in the training data subset. When this network and threshold were used with the testing data subset, it had an accuracy of 60.3%. Like several of the other networks, this network is an example of how training can produce, in some circumstances, similar optimization in different network designs; however, not all networks will be able to be optimized to the highest level of performance. 

### 6.10. Tenth Network Implementation and Results

**Figure 12:**
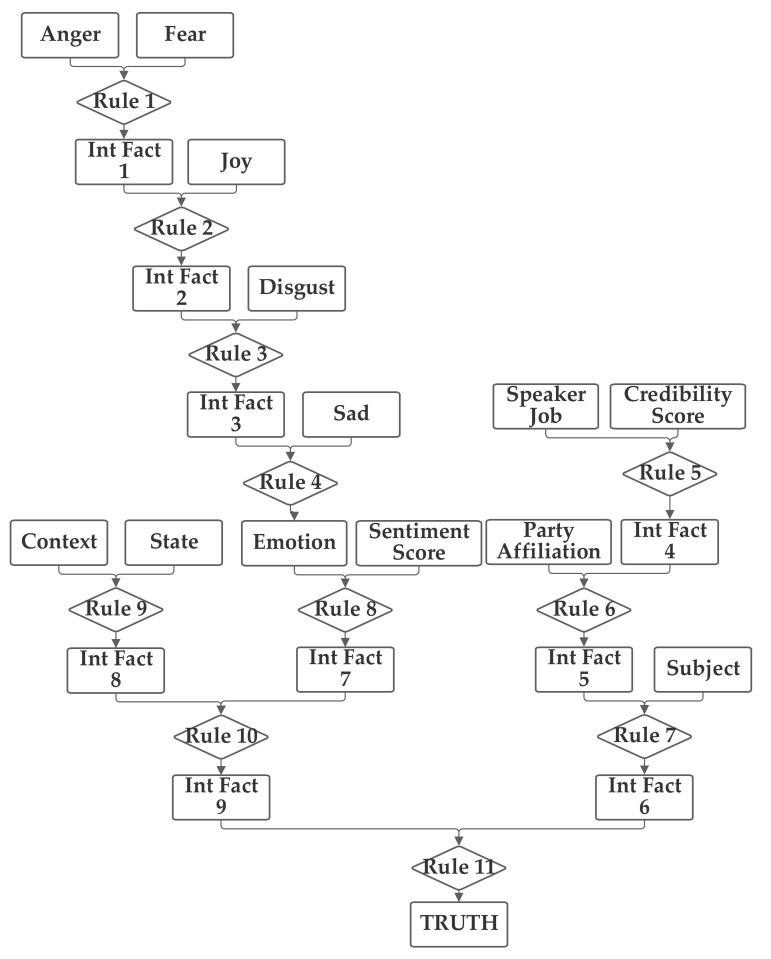
Depiction of Network 10.

In the tenth network, presented in Figure 12, the emotion value (based on the five emotion-related inputs) and the sentiment input are grouped together. The context and state values are grouped together as are (separately) the job, credibility, party, and subject inputs.

The tenth network, tied with networks 1, 3, 6, 7 and 8 (and one more subsequently discussed) to produce the highest accuracy level of 62.4%. Like with the previous networks, it was trained with both 1 and 100 training epochs, and the 1 epoch of training results are now discussed. With the first 1000 data records in the training subset, the normalization was evaluated and 21.7% were matched to the correct one of the six categories without threshold normalization. The applicable threshold value was again computed to be 0.16, using the data from the training dataset. With this threshold, it accurately classified 63.2% of the records in the training data subset. When this network and threshold were used with the testing data subset, it had an accuracy of 62.4%, again showing how training can produce, in some circumstances, similar optimization in different network designs. Like with network eight, this network further demonstrates the importance of the threshold value for scaling, as it shows how different network configurations can alter the magnitude of the output of the truth value, while still producing a similar logical result.

### 6.11. Eleventh Network Implementation and Results

**Figure 13:**
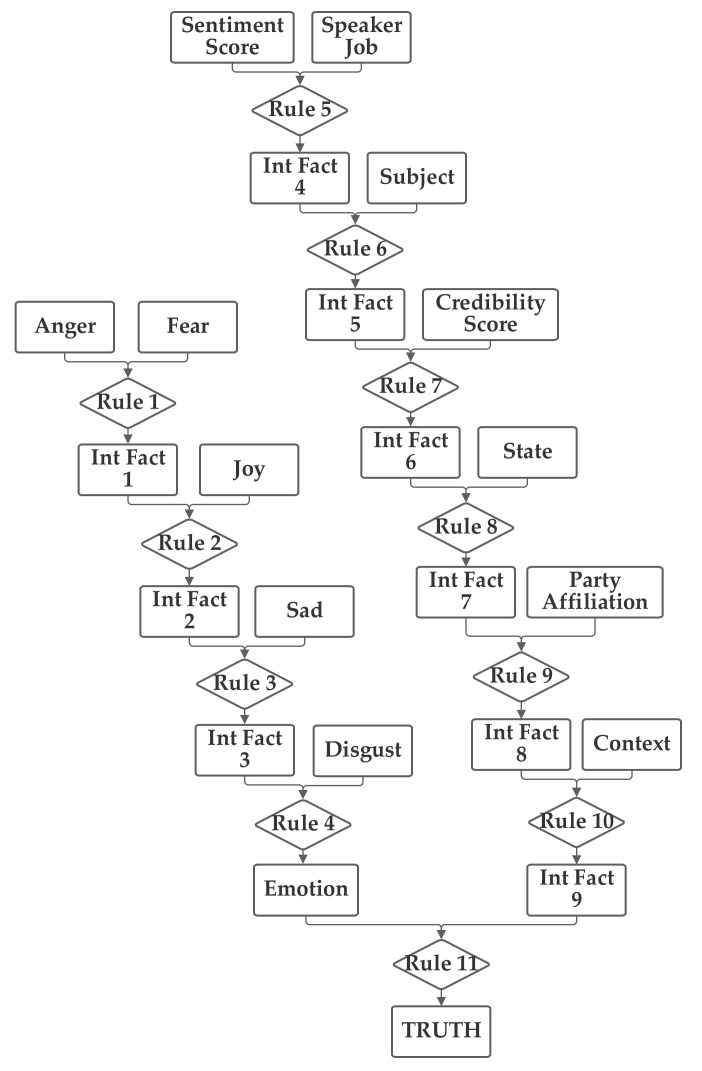
Depiction of Network 11.

In this network, shown in Figure 13, the emotion score is combined, at the end of the network, with all of the other inputs to produce the truthfulness output fact. The sentiment, context, state, job, credibility, party, and subject inputs are grouped together.

Like with the previous networks, the eleventh network was trained with both 1 and 100 training epochs, and the 1 epoch of training results are now discussed. With the first 1000 data records in the training subset, the normalization was evaluated and 21.0% were matched to the correct one of the six categories without threshold normalization. The applicable threshold value was again computed to be 0.11, using the data from the training dataset. With this threshold, it accurately classified 62.0% of the records in the training data subset. When this network and threshold were used with the testing data subset, it had an accuracy of 58.8%. This is the second worst result of the twelve networks, making it a demonstration of how network configuration clearly can impact performance and how training cannot always overcome network design decisions.

### 6.12. Twelvth Network Implementation and Results

**Figure 14:**
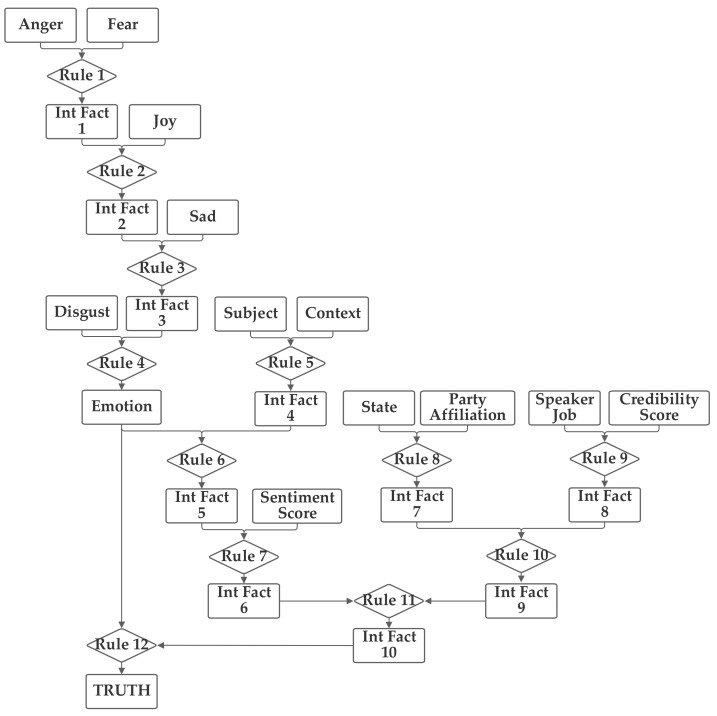
Depiction of Network 12.

The twelfth network was designed quite similarly to the first one. However, in the twelfth network, the emotion fact (which is based on the five emotion inputs) serves as an input to two rules. It contributes to an intermediate fact, along with the sentiment score, and directly to the final rule that produces the output truthfulness fact. The gradient descent trained expert system is designed to support facts serving as inputs to multiple rules. Due to the limited number of inputs, this capability hasn’t been used much in this study; however, this network demonstrates the capability. The development of more complex networks serves as a key area of future work for the deceptive content detection project, in addition to exploring the use of other textual analysis pre-processing techniques. This network is an example of a slight increase in complexity. The twelfth network is shown in Figure 14.

The tenth network, tied with networks 1, 3, 6, 7, 8, and 10 to produce the highest accuracy level of 62.4%. Like with the previous networks, it was trained with both 1 and 100 training epochs, and the 1 epoch of training results are now discussed. With the first 1000 data records in the training subset, the normalization was evaluated and 22.1% were matched to the correct one of the six categories without threshold normalization. The applicable threshold value was again computed to be 0.14, using the data from the training dataset. With this threshold, it accurately classified 63.2% of the records in the training data subset. When this network and threshold were used with the testing data subset, it had an accuracy of 62.4%, again showing—even with the additional complexity of the use of the emotion fact as the input to two rules—how training can produce, in some circumstances, similar optimization in different network designs. It once again demonstrates the importance of the threshold value for scaling, as well.

### 6.13. Analysis of Results

The previous sub-sections have presented and analyzed twelve rule-fact networks that were developed for the purposes of deceptive content identification. Each section presented the results from 1 epoch of training for the network, though it was noted that the system was tested with 100 epochs of training, as well. Table 3 presents the result from both 1 training epoch and 100 epochs for each of the twelve networks. As is obvious from this table, the two levels of training performed very similarly, with only networks 2 and 11 showing a difference in results. As network 2’s performance decreases and network 1’s performance increases with 100 epochs of training (as compared to 1 epoch), neither level of training can be said to consistently outperform the other.

Similarly, Table 4 compares the thresholds selected, due to their superior performance, for the 12 networks under 1 and 100 epochs of training. It also presents the performance of the threshold for the training data for each network. It is notable that for all but one of the 12 networks, the threshold value is the same for both the 1 and 100 epochs of training. Further, for all of the networks, the performance with the training data was the same between the 1 and 100 epochs of training. This is further evidence of the lack of additional benefit produced by the additional training, for this particular application. Clearly, the use of over 10,000 training records was, by itself, sufficient to train the network without the need for multiple iterations of training with each record.

The one difference between the two training levels, the threshold values for network 1, is indicative of there being two equivalent threshold levels (in terms of the results that they produce). This is supported by the equivalent results for performance with both the training and actual testing data for the two levels of training.

Overall, the analysis of the twelve networks has demonstrated that network design changes can have a notable impact on system performance, as the performance difference between the best and worst performing networks was approximately 14%. However, it also showed that, in some cases, changes to network design can be immaterial as the training process can optimize them similarly to other networks.

The importance of the normalization threshold mechanism was also demonstrated, as all of the networks tended to reduce the output values significantly from the normalized target values. Notably, the output values ranged between 0.11 and 0.25, so the impact was different on a network-by-network basis, even though the logical results were quite similar (or the same) in many cases. 

### 6.14. Network Implementation Design Process

**Figure 15:**
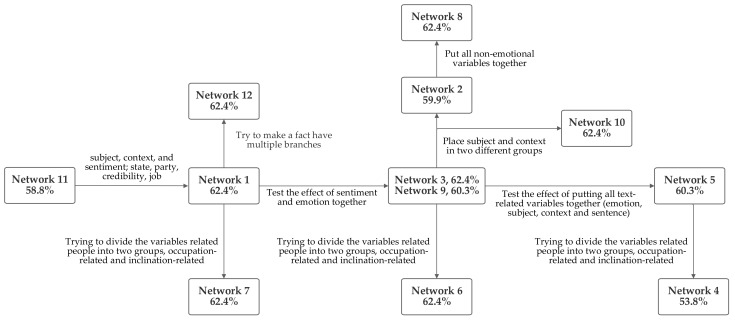
Network Changes and Results.

While Section 5.1 and Section 5.2 have presented the twelve networks that were evaluated and described their particular characteristics, this section focuses, briefly, on the similarities and differences between them. This, thus, facilitates a discussion of the design process that was used in creating the networks. Figure 15 depicts this visually and states the differences between adjacent networks designs. It also lists the accuracy level produced by each.

Multiple ideas for grouping the inputs were considered, as were discussed in Section 6.1, Section 6.2, Section 6.3, Section 6.4, Section 6.5, Section 6.6, Section 6.7, Section 6.8, Section 6.9, Section 6.10, Section 6.11 and Section 6.12. Since sentiment and emotion have conceptual similarities, they were grouped together, in some circumstances. Another design concept was grouping the inputs related to the text content together. This included the subject and context inputs, as well, in some cases, as the sentiment and emotion values that were produced from textual analysis. In some cases, speaker-related inputs were grouped. These included jobs, state, credibility, and party affiliation. All of these groupings are included in several of the networks. Notably, the network that was first arrived at through logical analysis, network 1, was one of the several networks which tied for producing top performance.

The analysis of the Figure 15 diagram reveals some patterns. The accuracy rate decrease from network 1, going rightwards, suggests that the emotion inputs produce higher accuracy when grouped. Another pattern of decreases suggests that accuracy decreases when the person-related inputs are divided into two groups, suggesting that the person-related inputs produce a higher level of accuracy when grouped together. However, comparing networks 2 and 8 suggests that grouping non-emotional factors together may harm accuracy. This suggests that it is better to classify non-emotional factors as being text-related or person-related. To evaluate whether putting all of the non-emotion related variables together might produce higher accuracy, network 11 was tested; however, this approach did not increase performance. Network 12 was designed to explore the impact of having facts serve as inputs to multiple rules. Thus, in network 12, the emotion value is included in a text-related grouping as well as being included at the end of the network with the text- and person-related groups to produce the output fact. However, this did not aid system performance.

Based on the foregoing, grouping the emotion and sentiment inputs together, grouping all of the human-related inputs together and grouping all of the text-related inputs together would be expected to result in the highest accuracy. This is the design foundation of network 1. Given that network 1 was the network initially arrived at by the logical analysis of the interrelationships between the inputs, the fact that the pattern of performance between the different networks suggests that its characteristics should perform the best serves to validate key design decisions.

## 7. Comparison to the Results of Prior Work

Comparing the performance of the system presented herein to prior work shows that it outperforms several prior implementations, while underperforming others. Problematically, several prior studies that have used the LIAR dataset have failed to fully describe their method of analysis, making a direct comparison problematic. Long, et al. [52] demonstrated an accuracy of 27% using a convolutional neural network and reached 41.5% when combining several techniques. It is not clear, from their paper, whether they are making a true versus false classification or evaluating classification into the six truthfulness levels. Yang, et al. [53] reported accuracy levels between 58.6% and 75.9% using techniques such as “majority voting” and an “unsupervised fake news detection framework,” using only a subset of 322 of the 12,800 LIAR records with particular characteristics. 

Singh [36] obtains results ranging between 45.83% and 59.82% accuracy using four different vector space representations and three different types of neural networks. Upadhayay and Behzadan [42] developed and used the additional sentiment fields in the Sentimental LIAR dataset, which are based on natural language processing, as well as a “bidirectional encoder representations from transformer” system (which is not utilized in this work) and achieved accuracy levels ranging from 55.46% to 70.00%.

The accuracy levels presented herein outperform many of the examples of prior work (which are summarized in Table 5) and fall within the range of the performance of Upadhayay and Behzadan’s system. Notably, this paper uses the same evaluation mechanism as Upadhayay and Behzadan did, so this is the most direct comparison of those discussed.

The system described herein, thus, is able to produce results that outperform one of Upadhayay and Behzadan’s techniques, while underperforming or approximately equivalently performing with several others without using the additional computationally expensive “bidirectional encoder representations from transformer” system.

These results are, thus, notable in comparison to prior work as they show that the sentiment processing, by itself, can produce results close to those performed with the additional “bidirectional encoder representations from transformer” system. Additionally, the results demonstrate the efficacy of the gradient descent trained expert system, in one of its first applications to a real-world problem. Unlike the neural networks and other techniques used by many studies, the gradient descent trained expert system technique is new and still being explored to identify how to best implement it across different problem types and with data with different types of characteristics.

## 8. Limitations, Other Uses and Potential Enhancements

The technique used herein is inherently limited by the manual process of the creation of the networks. To utilize the machine learning trained expert system for other applications, it is necessary to gain an understanding of the application area and to design and validate a logical network for the application. The work presented herein, in particular, has shown that multiple similar implementations of an application’s logical interrelations may perform differently, so it will likely actually be necessary to make several networks and evaluate their comparative performance. The overall operational performance is limited by the accuracy of the human-generated networks, and it is possible that an optimal network may never be realized. The manual nature of the network creation is what protects against the learning of invalid, potentially illegal and non-causal relationships; however, it makes the process of implementation far more manually intensive and time consuming than the use of a neural network for a given application.

Additionally, some logical constructs—particularly those that do not satisfy the transitive property of multiplication and division—cannot be effectively represented by the network structures utilized with this technique. Potentially these could be implemented through the implementation of multiple sub-systems whose networks are interrelated outside of the machine learning trained expert system environment.

Future work can potentially enhance the system’s performance through the automation of network creation (while ensuring that human control is maintained to prevent problematic associations being learned) and the implementation of other node relationships beyond multiplication-based ones.

## 9. Conclusions and Future Work

Among the twelve networks, network 1 was the best performer, both in terms of accuracy and in terms of the difference between predicted and actual values. Notably, network 1 was the network that was initially created based on the most apparent logical interrelationship between the different inputs.

Several patterns were also discussed in Section 6.14, providing some knowledge about the application domain itself. It was shown that the emotion inputs produce higher accuracy levels when placed into groups and that grouping the person-related inputs also increases accuracy. Grouping all of non-emotional factors, conversely decreased accuracy. These observations led to the conclusion that grouping emotion and sentiment together, grouping all of the human-related inputs together, and grouping all of the text-related inputs together will result in the highest accuracy rate, which provides a conceptual explanation for the performance of network 1. Given that this grouping strategy makes logical sense, it is a demonstration of the efficacy of the gradient descent trained expert system approach.

The networks presented herein outperformed the results of several prior studies that used the LIAR dataset (albeit, with some question regarding the exact evaluation procedure used by these studies). They also performed within the range of performance of Upadhayay and Behzadan’s [42] study, which introduced the Sentimental LIAR dataset. Notably, this similar performance was without using the additional textual analysis from the “bidirectional encoder representations from transformer” used by Upadhayay and Behzadan.

Beyond the particular deceptive content identification performance of this system, the perhaps more notable contribution of this paper is the demonstration of the efficacy of the gradient descent trained expert systems technique to a real-world application. The fact that the newly developed system performs similarly to more established and analyzed techniques is a demonstrable validation of the new system. Additionally, the correlation between the most logical network design and strongest performance is notable.

Clearly, there are a number of directions for future work. As a new technique, the gradient descent trained expert system algorithm will benefit from further analysis and potential refinement. The areas for enhancement mentioned in the previous section could be pursued. Additionally, techniques for identifying and measuring relationships between inputs without necessitating a network to be built and evaluated could enhance system development speed and, thus, would be a useful future area of work. Also, building in normalization mechanisms could be demonstrably beneficial. 

In terms of this particular study, additional network designs could be evaluated. Given that an infinite number of networks are possible, there are numerous additional ones beyond the twelve discussed herein that could be explored. In particular, additional more complex networks could be evaluated. The number of possible networks is constrained, somewhat, by close associations between some variables; however, associations or categorization does not fundamentally alter the network structure and overreliance on this simplification may result in error where these simplifications don’t hold true. The use of additional inputs, such as the “bidirectional encoder representations from transformer” used by Upadhayay and Behzadan, could also be evaluated to see what impact they may have in enhancing system performance.

## Figures and Tables

**Table 1:** Network Inputs (input names are from the Sentimental LIAR dataset [42]).

Input	Description	Rationale
subject	the subject of the statement (such as various important and common political issues)	provides background information of the statement
context	the platform where the statement was delivered and the type of statement	provides background information of the statement
sentiment score	indicates the polarity of the statement, whether it is positive, negative, or neutral	characterizes the text itself
state	U.S. state that the speaker is from	provides background information on the speaker
party affiliation	speaker’s party affiliation	provides background information on the speaker
credibility score	reflects how likely the speaker is to tell the truth based on past statements	provides background information on the speaker
speaker job	the job held by the speaker	provides background information on the speaker
anger	the proportion of anger in the statement	characterizes the text itself
fear	the proportion of fear in the statement	characterizes the text itself
joy	the proportion of joy in the statement	characterizes the text itself
disgust	the proportion of disgust in the statement	characterizes the text itself
sad	the proportion of sad in the statement	characterizes the text itself

**Table 2:** Example Data (input names and example data from the Sentimental LIAR dataset [42]).

Input	Example Data
subject	abortion energy health-care
context	a news release an interview on CNN a tweet
state	Texas Virginia Illinois
party affiliation	Republican Democrat independent
speaker job	state representative state delegate president

**Table 3:** Network accuracy results for 1 and 100 training epochs.

	1 Epoch	100 Epochs
Network 1	62.4%	62.4%
Network 2	59.9%	57.2%
Network 3	62.4%	62.4%
Network 4	53.8%	53.8%
Network 5	60.3%	60.3%
Network 6	62.4%	62.4%
Network 7	62.4%	62.4%
Network 8	62.4%	62.4%
Network 9	60.3%	60.3%
Network 10	62.4%	62.4%
Network 11	58.8%	61.2%
Network 12	62.4%	62.4%

**Table 4:** Threshold values and performance with training data for 1 and 100 training epochs.

	1 Epoch		100 Epochs	
	Threshold	Train Data	Threshold	Train Data
Network 1	0.11	63.2%	0.25	63.2%
Network 2	0.11	60.1%	0.11	60.1%
Network 3	0.15	63.2%	0.15	63.2%
Network 4	0.11	54.1%	0.11	54.1%
Network 5	0.11	60.7%	0.11	60.7%
Network 6	0.11	63.2%	0.11	63.2%
Network 7	0.14	63.2%	0.14	63.2%
Network 8	0.17	63.2%	0.17	63.2%
Network 9	0.11	60.7%	0.11	60.7%
Network 10	0.16	63.2%	0.16	63.2%
Network 11	0.11	62.0%	0.11	62.0%
Network 12	0.14	63.2%	0.14	63.2%

**Table 5:** Comparison of Different Prior Approaches.

Approach	Best Accuracy *
Long, et al. [52]—Conventional Neural Network	27%
Long, et al. [52]—multiple techniques combined	41.5%
Yang, et al. [53]	75.9%
Singh [36]	59.82%
Upadhayay and Behzadan [42]	70%
System described herein	62.4%

* Due to ambiguities in reporting technique descriptions and limitations of study results descriptions, it is possible that results may differ in terms accuracy calculation technique.

## Data Availability

This manuscript analyzed data that is already publicly available. No new dataset was generated from this research.
